# Detection of latent tuberculosis infection in patients prior to and following heart transplantation

**DOI:** 10.3389/fmed.2026.1878586

**Published:** 2026-07-15

**Authors:** Starshinova Anna, Sabirova Adilia, Fedotov Petr, Musaeva Bulgun, Sharipov Raul, Kudryavtsev Igor, Artem Rubinstein, Vasilyeva Elena, Stabrova Elena, Kudlay Dmitry

**Affiliations:** 1 Almazov National Research Medical Center, St. Petersburg, Russia; 2 Faculty of Mathematics and Computer Science, Saint Petersburg State University, St. Petersburg, Russia; 3 Medical Department, Bashkir State Medical University of the Ministry of Health of the Russian Federation, Ufa, Russia; 4 Institute of Experimental Medicine, St. Petersburg, Russia; 5 Department of Pharmacology, Institute of Pharmacy, I.M. Sechenov First Moscow State Medical University, Moscow, Russia; 6 FSBI SSC Institute of Immunology FMBA of Russia, Moscow, Russia; 7 Faculty of Bioengineering and Bioinformatics, Lomonosov Moscow State University, Moscow, Russia

**Keywords:** diaskintest, heart transplantation, IGRA, immunodiagnostics, immunosuppression, latent tuberculosis infection, QuantiFERON^®^ -TB Gold ELISA

## Abstract

**Background:**

Early identification of latent tuberculosis infection (LTBI) is essential for preventing active tuberculosis in heart transplant candidates and recipients, particularly in the context of immunosuppressive therapy.

**Aim:**

To assess and compare the prevalence of LTBI in patients awaiting heart transplantation and in heart transplant recipients using modern immunodiagnostic methods.

**Methods:**

The prospective study was conducted between 2025 and 2026 and included patients listed for heart transplantation at the Almazov National Medical Research Centre. Patients were divided into two main groups according to whether heart transplantation was performed was conducted to evaluate immunodiagnostic: I group - patients prior to heart transplantation (*n* = 14) and the II group - in heart transplant recipients (*n* = 28). All patients included on the heart transplantation waiting list underwent a comprehensive assessment, which included detailed immunological testing. LTBI was assessed using (QuantiFERON^®^-TB Gold ELISA) and/or a recombinant tuberculosis antigen skin test (Diaskintest). Statistical analysis was performed using Pearson’s χ^2^ test, with significance set at *p* < 0.05.

**Results:**

LTBI prevalence was significantly higher in heart transplant recipients (53.6%) compared with controls (5.8%; χ^2^ = 23.366, *p* = 0.001). In pre-transplant patients, LTBI prevalence was 35.7%, also significantly exceeding that of controls (χ^2^ = 9.058, *p* = 0.003). The higher LTBI rates in both patient groups may reflect immune dysregulation associated with chronic heart failure and the effects of immunosuppressive therapy. Although LTBI prevalence was higher in transplant recipients than in pre-transplant patients, the difference was not statistically significant.

**Conclusion:**

Heart transplant recipients demonstrate the highest prevalence of LTBI, followed by patients awaiting transplantation, both markedly exceeding that observed in healthy individuals. These findings indicate an elevated risk of active tuberculosis in this population and support the importance of routine LTBI screening and preventive therapy.

## Introduction

Early identification of tuberculosis infection is a key component in preventing active tuberculosis in solid organ transplant recipients, particularly at the pre-transplant stage. According to the World Health Organization guidelines (1), candidates for organ transplantation are classified as a high-risk group for tuberculosis, necessitating systematic screening using modern immunodiagnostic methods (1).

The true prevalence of latent tuberculosis infection (LTBI) among transplant candidates remains uncertain. Previous studies have reported LTBI prevalence rates of 31% in kidney transplant candidates, 25% in liver transplant candidates, and 13% in haematopoietic stem cell transplant candidates based on interferon-gamma release assays (IGRAs) (2). Most published studies and systematic reviews combine heart transplant recipients with other solid organ transplant populations, limiting the availability of heart-specific epidemiological data. Nevertheless, heart transplant recipients represent a particularly vulnerable population because of advanced heart failure, multiple comorbidities, and the requirement for intensive and prolonged immunosuppressive therapy after transplantation. Reactivation of latent tuberculosis in this setting is associated with substantial morbidity and mortality (3, 4).

Given the limited evidence regarding LTBI prevalence and screening performance in heart transplant candidates and recipients, particularly in countries with a high tuberculosis burden, further studies are needed to optimize pre-transplant screening strategies and identify patients at increased risk of post-transplant tuberculosis (5).

In addition, recent data from transplant recipients indicate LTBI prevalence as high as 46.9%, highlighting the critical importance of pre-transplant risk assessment.

In the general population, the lifetime risk of developing active tuberculosis in individuals infected with *Mycobacterium tuberculosis* is estimated at 5–10%, most commonly within the first 5 years after infection (6). However, this risk is substantially increased in individuals with impaired immune function. Among solid organ transplant recipients, the incidence of tuberculosis is significantly higher than in the general population, largely due to immunosuppressive therapy, which disrupts T-cell-mediated immunity (7).

Modern immunosuppressive regimens, including calcineurin inhibitors, antimetabolites, mTOR inhibitors, and glucocorticosteroids, effectively prevent graft rejection but contribute to immune dysregulation, thereby increasing susceptibility to opportunistic infections (6, 7). In this context, tuberculosis in transplant recipients most commonly results from reactivation of LTBI and is often characterized by atypical clinical presentation, delayed diagnosis, and a higher frequency of disseminated and extrapulmonary disease (8–10). These factors, together with drug–drug interactions between anti-tuberculosis and immunosuppressive agents, complicate management and may adversely affect transplant outcomes (11, 12). Despite advances in diagnostics, challenges remain in both the timely detection of LTBI and the selection of safe and effective treatment regimens in transplant populations. This is particularly relevant given the potential toxicity and interactions of anti-tuberculosis drugs, as well as the limited therapeutic options in cases of drug-resistant tuberculosis (13, 14).

Therefore, the detection of LTBI in patients with chronic heart failure undergoing evaluation for heart transplantation, as well as in heart transplant recipients, is essential for the timely initiation of preventive therapy, which may reduce the risk of active tuberculosis, improve transplant outcomes, and enhance patient survival.

## Aim of the study

To assess the frequency of positive latent tuberculosis infection (LTBI) screening results in patients prior to heart transplantation and in heart transplant recipients using contemporary immunodiagnostic methods and to explore potential differences between these populations.

## Materials of the study

### Study design and participants

**FIGURE 1:**
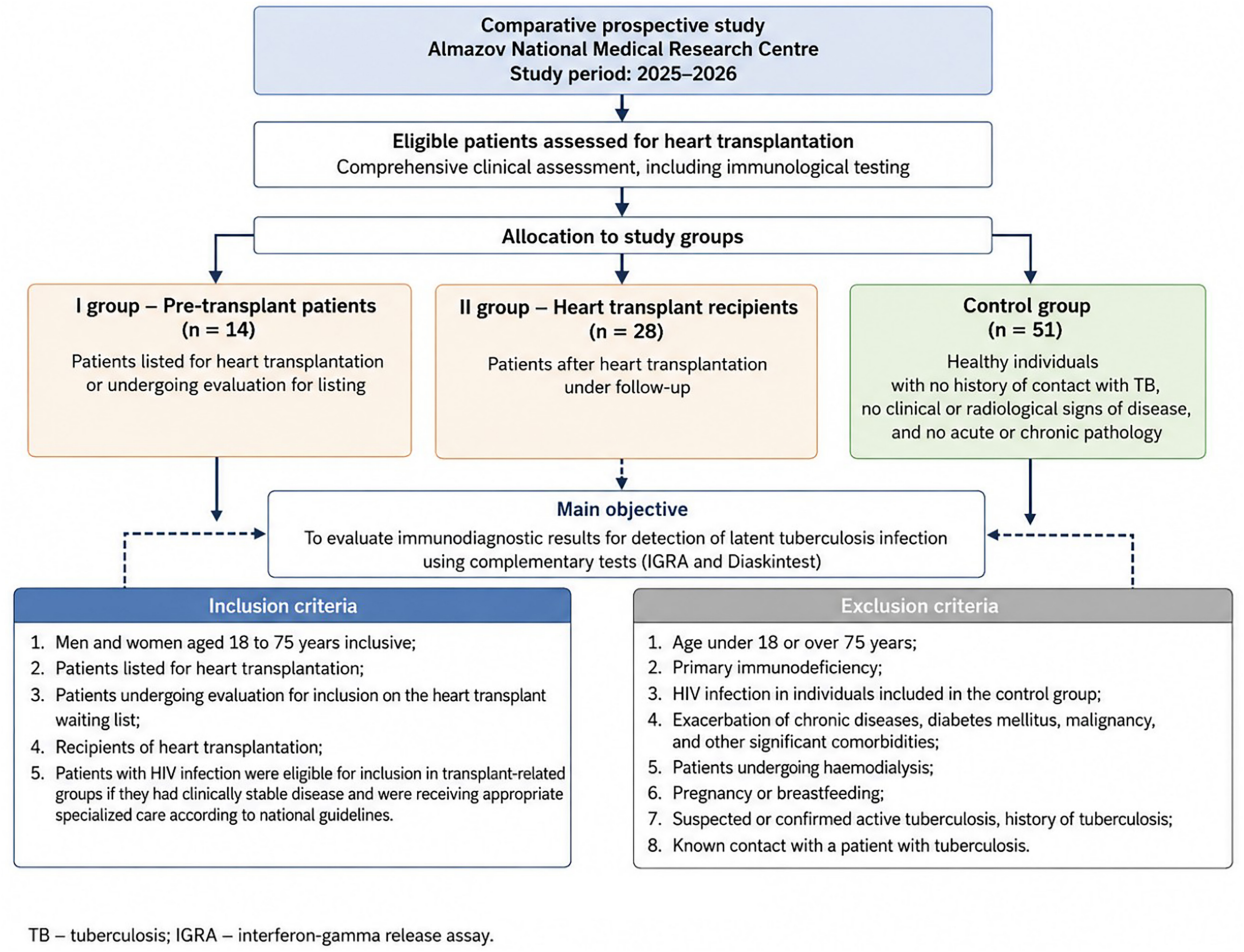
Design of the study. TB, tuberculosis; IGRA, interferon-gamma release assays.

The comparative prospective study was conducted between 2025 and 2026 and included patients listed for heart transplantation at the Almazov National Medical Research Centre (Figure 1).

Patients were divided into two main groups according to whether heart transplantation was performed was conducted to evaluate immunodiagnostic: I group - patients prior to heart transplantation (*n* = 14) and the II group - in heart transplant recipients (*n* = 28). All patients included on the heart transplantation waiting list underwent a comprehensive assessment, which included detailed immunological testing. The control group (*n* = 51) consisted of healthy individuals with no history of contact with tuberculosis, no clinical or radiological signs of disease, and no acute or chronic pathology, including during exacerbation. The distribution of study participants across groups was performed in accordance with.

Inclusion criteria were:

1) men and women aged 18 to 75 years inclusive;
2) patients listed for heart transplantation;
3) patients undergoing evaluation for inclusion on the heart transplant waiting list;
4) recipients of heart transplantation.
5) Patients with HIV infection were eligible for inclusion in transplant-related groups if they had clinically stable disease and were receiving appropriate specialized care according to national guidelines.

Exclusion criteria were:

(1) age under 18 or over 75 years;
(2) primary immunodeficiency;
(3) HIV infection in individuals included in the control group;
(4) exacerbation of chronic diseases, diabetes mellitus, malignancy, and other significant comorbidities;
(5) patients undergoing haemodialysis;
(6) pregnancy or breastfeeding;
(7) suspected or confirmed active tuberculosis, history of tuberculosis;
(8) known contact with a patient with tuberculosis.

Male predominance was observed in both the pre-transplant and heart transplant recipient groups (64.3% [*n* = 9; 95% CI 39.19–89.39] and 67.9% [*n* = 19; 95% CI 50.56–85.16], respectively), whereas females predominated in the control group (62.7% [*n* = 32; 95% CI 49.48–76.01]) (Table 1).

**TABLE 1:** Characteristics of patients in the study groups by gender and age.

Groups of patients	Men	Women	Age
	% (*n*)	95% CI	% (*n*)
Pre-transplant patients (*n* = 14)	64.3 (9)	[39.19; 89.39]	35.7 (5)
Heart transplant recipients (*n* = 28)	67.9 (19)	[50.56; 85.16]	32.1 (9)
Healthy controls (*n* = 51)	37.3 (19)	[23.99; 50.52]	62.7 (32)

The mean age of patients in the comparison groups was 52.3 years (95% CI 45.20–59.40) and 53.9 years (95% CI 49.29–58.51), respectively, compared with 29.2 years (95% CI 26.79–31.61) in the control group. Thus, the pre-transplant and transplant recipient groups were comparable in terms of age and sex distribution.

The differences in age and sex distribution between patient groups and healthy controls reflect the real-world epidemiology of advanced heart failure and heart transplantation, where older age and male predominance are commonly observed. These differences should be considered when interpreting intergroup comparisons because demographic characteristics may influence LTBI screening outcomes.

The pre-transplant group consisted of patients with clinical and instrumental manifestations of chronic heart failure. In the majority of patients (64.3% [*n* = 9; 95% CI 39.19–89.39]), the New York Heart Association (NYHA) functional class was assessed as Class II, characterized by symptoms such as dyspnoea, palpitations, and fatigue during ordinary physical activity.

A total of 85.7% (*n* = 12; 95% CI 58.81–97.24) of patients were undergoing inpatient cardiology evaluation to determine eligibility for inclusion on the transplant waiting list. Of these, 8.3% (*n* = 1; 95% CI 0.21–38.48) declined listing. Overall, 7.1% (*n* = 1; 95% CI 1.27–31.47) were fully evaluated and included on the waiting list, while another 7.1% (*n* = 1; 95% CI 1.27–31.47) were subsequently excluded due to technical contraindications identified by a cardiac surgical board.

All patients with chronic heart failure and reduced ejection fraction received guideline-directed quadruple therapy, including angiotensin receptor–neprilysin inhibitors, beta-blockers, mineralocorticoid receptor antagonists, and sodium–glucose cotransporter 2 inhibitors, along with additional supportive therapies. Implantable cardioverter-defibrillator placement was planned in 14.3% of patients (*n* = 2; 95% CI 4.01–39.94) due to life-threatening arrhythmias. One patient (7.1%) received oral hypoglycaemic therapy for diabetes mellitus. Another patient (7.1%) had been previously evaluated by a phthisiatrician and was receiving preventive therapy for LTBI (isoniazid and rifampicin).

### Clinical characteristics of heart transplant recipients

In the transplant recipient group, 7.1% of patients (*n* = 2; 95% CI 0.90–23.73) had HIV infection. Their inclusion was justified by stable remission under antiretroviral therapy, characterized by suppressed viral load, adequate CD4+ T-cell counts, good adherence, and regular infectious disease follow-up.

According to the National Immunization Programme of the Russian Federation, BCG vaccination is routinely administered during the neonatal period, with revaccination historically performed in selected age groups. Therefore, all participants had received BCG vaccination in childhood. Information regarding individual vaccination status was collected from available medical records when possible. However, Diaskintest^®^ contains specific recombinant antigens ESAT-6 and CFP-10, its diagnostic performance is not significantly influenced by previous BCG vaccination.

Among transplant recipients, 3.6% (*n* = 1; 95% CI 0.63–17.71) had a history of hypertrophic cardiomyopathy progressing to dilated cardiomyopathy secondary to cardiac sarcoidosis.

All transplant recipients were under lifelong multidisciplinary follow-up involving transplant specialists, cardiologists, cardiac surgeons, and clinical pharmacologists to monitor graft rejection, coronary allograft vasculopathy, malignancies, infectious complications, hypertension, nephropathy, diabetes mellitus, and psychological conditions.

Immunosuppressive regimens included tacrolimus in 92.6% (*n* = 26), mycophenolate mofetil in 57.1% (*n* = 16), everolimus in 42.9% (*n* = 12), glucocorticosteroids in 32.1% (*n* = 9), and ciclosporin in 7.1% (*n* = 2). Patients receiving calcineurin inhibitors and/or mycophenolic acid derivatives underwent monthly therapeutic drug monitoring.

In addition to immunosuppressive therapy, patients received treatment for blood pressure control, lipid management, thrombosis prevention, supplementation (folic acid, calcium), and prophylaxis of oedema, osteoporosis, gastrointestinal lesions, and opportunistic infections. Antiretroviral therapy was administered in 7.1% of patients.

### Methods of the study

All participants underwent detailed medical history assessment, including evaluation of tuberculosis exposure. Standard diagnostic evaluation included chest radiography and immunological testing.

### Immunodiagnostic assessment

Diaskintest^®^ (Generium, Russian Federation) is a skin test based on recombinant tuberculosis allergen containing two Mycobacterium tuberculosis-specific antigens, ESAT-6 and CFP-10. These antigens are encoded within the RD1 genomic region and are absent from *Mycobacterium bovis* BCG strains and most environmental mycobacteria.

The test was administered intradermally in the middle third of the forearm according to the manufacturer’s instructions. The result was evaluated after 72 h by measuring the transverse diameter of induration. The test was interpreted as positive in the presence of any palpable induration, irrespective of size, and negative in the absence of induration.

Due to the absence of ESAT-6 and CFP-10 in BCG vaccine strains, the test demonstrates high specificity in BCG-vaccinated populations. However, limited cross-reactivity may occur with certain non-tuberculous mycobacterial species expressing homologous antigens.

Immunodiagnostic methods included IGRA test (QuantiFERON^®^-TB Gold ELISA (QFT)) and/or a recombinant tuberculosis antigen skin test (Diaskintest, Russia). In both study groups, all patients underwent IGRA testing. In the control group, 18 individuals underwent both tests, while 33 underwent only the Diaskintest.

The results of QFT and the Diaskintest were interpreted according to standard guidelines. Individuals with positive results underwent comprehensive evaluation to exclude active tuberculosis, including chest computed tomography.

### Statistical analysis

Statistical analysis and data visualization were performed using Microsoft Excel 2019, Statistica 8.0 (StatSoft, USA), and GraphPad Prism 4.0 (GraphPad Software Inc., USA).

The prevalence of LTBI was defined as the proportion of positive immunological test results. Statistical significance between groups was assessed using Pearson’s χ^2^ test, with *p* < 0.05 considered statistically significant.

Confidence intervals (95% CI) for continuous variables (age) were calculated using descriptive statistics and the one-sample Student’s *t*-test. For binomial variables (sex, test results). Given the exploratory nature of the study and the limited sample size, analyses were primarily descriptive. Pearson’s χ^2^ test was used to compare proportions between groups; however, the results should be interpreted cautiously because demographic and clinical differences between groups may act as confounding factors. Multivariable regression modelling was not performed because of the limited sample size and insufficient number of positive events, which could have led to unstable estimates and overfitting. Confidence intervals for binomial proportions were calculated using the Wilson score method, which provides more robust estimates for relatively small samples and low event frequencies. Confidence intervals were graphically represented using box plots in Microsoft Excel.

## Results

In the group of patients prior to heart transplantation, positive immunodiagnostic results were observed in 35.7% of cases (*n* = 5; 95% CI 16.18–61.40), which was significantly higher compared with healthy individuals (5.8%, *n* = 3; 95% CI 1.41–16.54; χ^2^ = 9.058, *p* = 0.003) (Table 2). This finding may reflect immune dysregulation associated with systemic inflammation in patients with chronic heart failure, as previously reported, as well as the relatively small sample size in this group (15, 16).

**TABLE 2:** Results of immunodiagnostic results in the study groups.

Group	Positive results % (*n*)	95% CI	Negative results % (*n*)	95% CI	χ ^2^	*p*-value
Pre-transplant patients (*n* = 14)	35.7 (5)	16.18–61.40	64.3 (9)	38.60–83.82	9.058	0.003
Heart transplant recipients (*n* = 28)	53.6 (15)	35.81–70.47	46.4 (13)	29.53–64.19	23.366	0.001
Healthy controls (*n* = 51)	5.8 (3)	1.41–16.54	94.2 (48)	83.46–98.59	–	–

Critical *χ*^2^ value = 3.841 (*p* < 0.05).

In heart transplant recipients, positive immunodiagnostic results were detected significantly more frequently than in the control group (53.6%, *n* = 15; 95% CI 35.81–70.47 vs. 5.8%, n = 3; χ^2^ = 23.366, *p* = 0.001). This likely reflects the immunosuppressive effects of therapeutic regimens, including tacrolimus, everolimus, mycophenolate mofetil, and other immunosuppressive agents.

No statistically significant differences were identified when comparing immunodiagnostic results between pre-transplant patients and heart transplant recipients (χ^2^ = 1.193, *p* = 0.275). However, these findings should be interpreted with caution, as the relatively small sample size in the pre-transplant group (*n* = 14) limits the statistical power and does not allow definitive conclusions regarding comparability between groups.

Notably, among the two individuals living with HIV included in the study, only one demonstrated a positive immunological test result.

**FIGURE 2:**
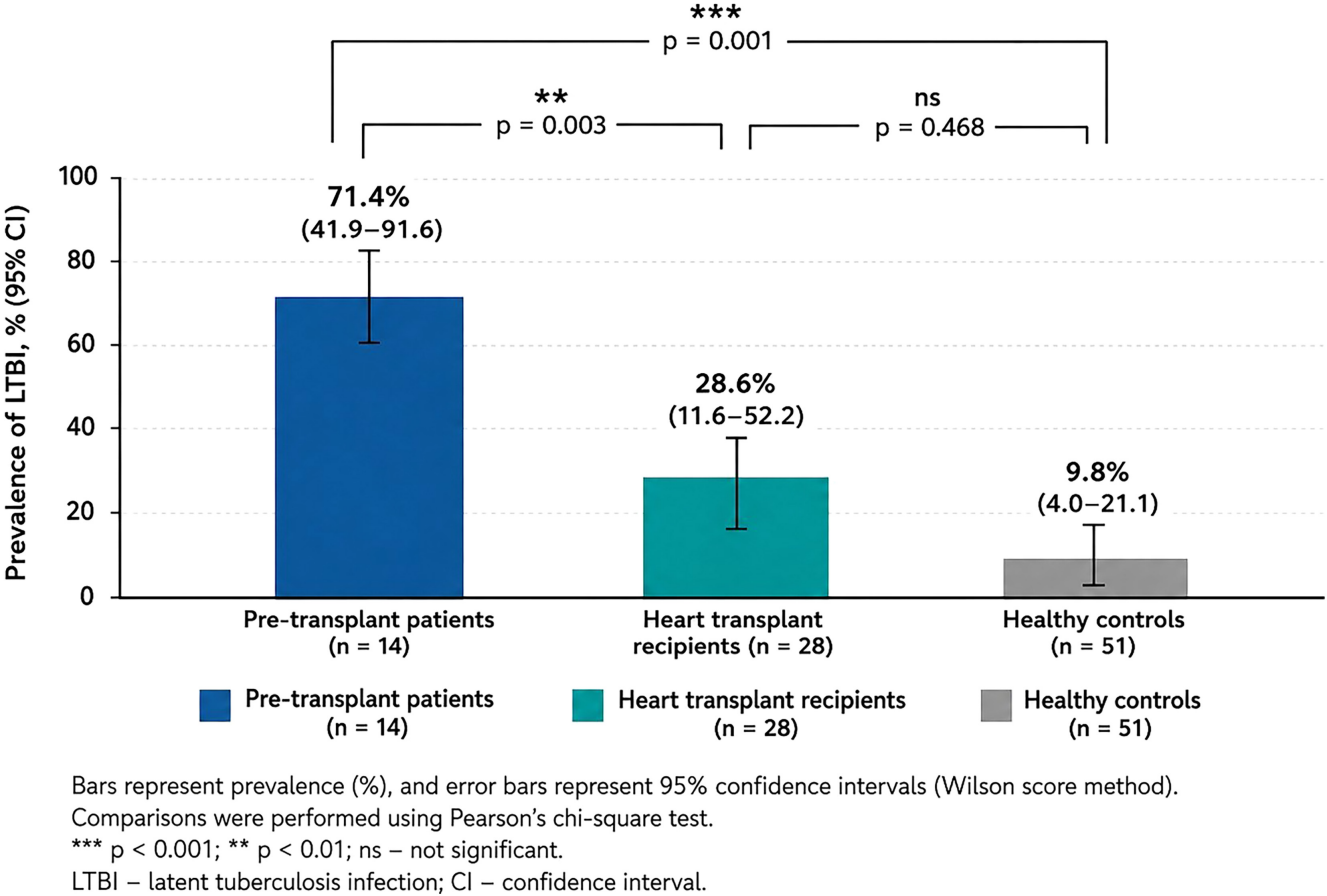
Prevalence of latent tuberculosis infection (LTBI) in study groups according to immunodiagnostic methods. Bars represent (%) and error bars represent 95% confidence intervals (Wilson score method). Comparisons were performed using Pearson’s chi-square test ***p<0.001; **p<0.01; ns, not significant; LTBI, latent tuberculosis infection; CI, confidence interval.

The proportion of positive results (53.6%) observed in heart transplant recipients (Figure 2) corresponds to the prevalence of latent tuberculosis infection (LTBI) in this group and is significantly higher than that observed in healthy individuals (5.8%).

The prevalence of LTBI in heart transplant recipients (53.6%) exceeded that in patients prior to transplantation (35.7%). However, the absence of statistically significant differences between these groups indicates the need for further investigation in larger cohorts.

## Discussion

The prevalence of latent tuberculosis infection (LTBI) among heart transplant recipients in our study reached 53.6%, which is approximately tenfold higher than that observed in healthy individuals from the general population (5.8%). This finding indicates a markedly increased risk of LTBI progression and subsequent development of active tuberculosis in this patient population (3, 17).

Our results are consistent with the nationwide study by Alpaydın et al., (3), who reported an LTBI prevalence of 46.9% (359/766) among solid organ transplant recipients based on the tuberculin skin test (TST) and interferon-gamma release assays (IGRAs). Notably, only 56.5% of LTBI-positive individuals received preventive therapy, highlighting the critical importance of identifying and managing LTBI prior to transplantation (18).

Despite advances in diagnostic approaches, the problem of LTBI detection and management in transplant recipients remains unresolved. Available data indicate that tuberculosis occurs 20–74% more frequently in transplant recipients compared to the general population. Subramanian and Theodoropoulos (18) emphasized the complexity of screening for both latent and active tuberculosis in transplant settings and highlighted the important role of IGRAs in improving diagnostic accuracy (19).

Another important aspect is the possibility of donor-derived tuberculosis transmission. Abad and Razonable (19) described 36 cases of donor-derived tuberculosis, with pulmonary forms predominantly observed in lung transplant recipients and extrapulmonary forms in recipients of liver, kidney, and heart transplants. These findings support the necessity of considering tuberculosis screening not only in recipients but also in organ donors (20, 21).

With the growing number of transplant procedures, including in pediatric populations, this issue becomes increasingly relevant. Pediatric transplant recipients are at higher risk of developing active tuberculosis and co-infections, including fungal infections. Sayyahfar et al., (21) demonstrated that the TST with 2 TU PPD-L had higher sensitivity compared to IGRA in pediatric heart transplant candidates, suggesting that both traditional and modern immunodiagnostic approaches may be complementary (21).

Heart transplant recipients are also at increased risk for a wide range of infectious diseases. Routine screening for HIV, hepatitis B and C, and implementation of prophylactic strategies against viral and fungal infections are essential. Simonenko et al., (22) reported a high incidence of COVID-19 (55%) in heart transplant recipients, further emphasizing the vulnerability of this population (22).

The prevalence of LTBI among patients awaiting heart transplantation in our study (35.7%) should be interpreted cautiously due to the limited sample size and wide confidence intervals. However, this relatively high prevalence may be explained by immune dysregulation associated with chronic heart failure (CHF) (23).

CHF is characterized by the inability of the heart to maintain adequate systemic perfusion. It is well established that CHF is associated with systemic inflammation, which plays a central role in disease pathogenesis and progression. Comorbid conditions such as diabetes mellitus, obesity, and chronic kidney disease contribute to a persistent low-grade inflammatory state. Additionally, activation of innate and adaptive immune responses, endothelial dysfunction, and inflammatory mediators originating from the gastrointestinal tract, spleen, and adipose tissue contribute to adverse cardiac remodeling (15, 24, 25).

Elevated levels of acute-phase proteins, including C-reactive protein, further support the presence of immune dysregulation. Redfield et al., (26) demonstrated elevated CRP levels in 57% of patients with CHF. Thus, patients with CHF awaiting heart transplantation exhibit persistent systemic inflammation and immune imbalance, which may predispose them to infectious diseases, including tuberculosis (26).

In addition, heart transplantation itself is associated with profound alterations in the adaptive immune system. Both pediatric and adult recipients demonstrate a marked reduction in circulating naïve CD4+ and CD8+ T cells. Studies have shown decreased levels of T-cell receptor excision circles (TRECs), reflecting impaired thymic output and reduced diversity of the T-cell receptor repertoire. Since naïve T cells are critical for recognizing novel antigens and initiating primary immune responses, their depletion may significantly compromise host defense against pathogens such as Mycobacterium tuberculosis (27).

Immunosuppressive therapy further contributes to immune dysfunction. Agents such as azathioprine and mycophenolate mofetil inhibit DNA synthesis and exert cytotoxic effects on proliferating lymphocytes, while calcineurin inhibitors (cyclosporine, tacrolimus) and mTOR inhibitors (sirolimus) suppress intracellular signaling pathways, T-cell activation, and clonal expansion. These mechanisms reduce immune competence and increase susceptibility to infections (28–30).

At the same time, recent evidence suggests that immune responses can be partially restored under certain conditions. Lazaro-Martin et al., (30) demonstrated that pediatric heart transplant recipients are capable of mounting an adequate immune response to hepatitis B revaccination, particularly when higher vaccine doses are used. Current guidelines recommend delaying vaccination for 3–6 months after transplantation, allowing stabilization of maintenance immunosuppressive therapy. Optimization of vaccination strategies may improve both *de novo* immune responses and maintenance of immunological memory (27).

Another important consideration is the possibility of false-negative immunodiagnostic results due to immune dysfunction. Tsai et al., (27) highlighted limitations of IGRA performance in patients with end-stage heart failure. Therefore, a combined diagnostic approach using both TST and IGRA may improve detection rates. Mardani et al., (17) reported only low agreement between these methods (*k* = 0.061), although IGRA demonstrated higher diagnostic value (31).

The interpretation of immunodiagnostic results is particularly challenging in HIV-positive transplant recipients. In our study, one of two HIV-positive patients had a negative result, which may represent a false-negative due to CD4+ T-cell depletion and anergy. In such cases, assays such as QuantiFERON^®^-TB Gold Plus, which evaluate both CD4+ and CD8+ T-cell responses, may offer improved sensitivity. Petruccioli et al., (28) demonstrated comparable performance of this assay in HIV-infected and uninfected individuals, likely due to compensatory CD8+ T-cell responses (32, 33).

Taken together, these findings highlight the importance of comprehensive immunodiagnostic screening for tuberculosis infection at the pre-transplant stage. Early identification of LTBI allows timely initiation of preventive therapy and specialized follow-up, ultimately reducing the risk of progression to active tuberculosis (34, 35).

Our findings also highlight the challenges associated with tuberculosis screening in immunocompromised populations. Conventional diagnostic approaches for tuberculosis, including immunological and microbiological methods, may demonstrate reduced sensitivity and limited diagnostic accuracy in certain clinical settings, particularly among patients with advanced chronic diseases and immunosuppression. Recent evidence suggests that emerging data-driven approaches, including machine learning-based models, may improve tuberculosis screening and risk stratification by integrating multiple clinical, laboratory, and imaging parameters. Although these approaches are not yet routinely applied in transplant medicine, they may represent a promising direction for future development of more accurate tuberculosis screening strategies in transplant candidates and recipients. These observations further support the need for comprehensive pre-transplant evaluation using complementary diagnostic methods rather than reliance on a single screening test (36).

### Diagnostic heterogeneity across LTBI studies

Comparisons of LTBI prevalence across studies should be interpreted cautiously because different diagnostic methods demonstrate variable sensitivity and specificity. The tuberculin skin test, interferon-gamma release assays (IGRAs), and recombinant antigen-based tests such as Diaskintest^®^ are based on different immunological principles and may yield substantially different prevalence estimates (37).

In populations with widespread BCG vaccination, the specificity of the tuberculin skin test may be reduced because of cross-reactivity with vaccine-induced immunity. In contrast, both IGRAs and Diaskintest^®^ utilize antigens absent from BCG strains, thereby improving specificity. Consequently, direct comparison of LTBI prevalence between studies using different diagnostic platforms should be performed with caution.

### Limitations of the study

Several limitations should be acknowledged. The sample size was relatively small, particularly in the pre-transplant group, which may have reduced statistical power. The study had a cross-sectional design and did not include longitudinal follow-up, preventing assessment of progression from LTBI to active tuberculosis. Multivariable regression modelling was not performed because of the limited sample size and number of positive cases. Detailed quantification of tuberculosis exposure intensity was not available. One limitation of the present study is the age difference between the control and transplant-related groups. Since cumulative exposure to *M. tuberculosis* increases with age, this discrepancy could potentially influence LTBI prevalence estimates. As cumulative exposure to *M. tuberculosis* may increase with age, differences in LTBI prevalence could be partially influenced by age-related factors. Future studies with age-matched cohorts and larger sample sizes are required to further clarify these associations. Although additional analyses did not demonstrate a significant association between age and test positivity, residual confounding cannot be fully excluded due to the sample size. The study was conducted in a limited number of centers within the Russian Federation, which may affect generalizability of the findings.

## Conclusion

The highest prevalence of LTBI, as determined by IGRA test and recombinant tuberculin allergen (Diaskintest) testing, was observed in heart transplant recipients (53.6%), exceeding that in patients awaiting transplantation (35.7%) and being significantly higher than in healthy individuals (5.8%). Heart transplant recipients demonstrated a numerically higher prevalence of LTBI compared with pre-transplant patients; however, this difference did not reach statistical significance. These findings indicate a substantial risk of progression to active tuberculosis and underscore the importance of early detection and preventive strategies in this patient population.

## Data Availability

The original contributions presented in this study are included in the article/supplementary material, further inquiries can be directed to the corresponding author.
